# Medical history, medication use and physical activity in adults in their eighth and ninth decade of life in the Hertfordshire Cohort Study

**DOI:** 10.17179/excli2022-4874

**Published:** 2022-04-19

**Authors:** Gregorio Bevilacqua, Jean Zhang, Camille Parsons, Faidra Laskou, Nicholas Fuggle, Cyrus Cooper, Elaine Dennison

**Affiliations:** 1 MRC Lifecourse Epidemiology Centre, Southampton SO16 6YD, UK; 2 NIHR Southampton Biomedical Research Centre, University of Southampton and University Hospital Southampton NHS Foundation Trust, Southampton, UK; 3 NIHR Oxford Biomedical Research Centre, University of Oxford, Oxford, UK; 4 Victoria University of Wellington, Wellington, New Zealand

**Keywords:** older, activity, medication, comorbidity, system

## Abstract

While there are many known health benefits to maintained physical activity levels in late adulthood, there have been very few studies that have considered relationships between morbidity profile and physical activity in the eighth decade of life. We studied 1097 participants, 555 men and 542 women from the Hertfordshire Cohort Study, a UK community based sample. Validated questionnaire based data were used to relate self-reported physical activity (PA) levels to medical history, and medication use. Regression analyses were adjusted for age, BMI, smoker status, alcohol consumption. The mean (SD) age of participants in the study was 80.2 (2.7) years for men and 80.2 (2.6) for women. A higher proportion of men (33.7 %) than women (24 %) were in the high activity score group. 20.8 % of female participants and 22.6 % male participants reported having no comorbid disease; 10.5 % men and 8.4 % women were taking no medication. Higher number of chronic conditions was associated with lower levels of PA [men (OR 0.73, 95 % CI 0.63-0.84, p<0.001); women (OR 0.74, 95 % CI 0.64-0.86, p<0.001)] as was being prescribed a higher number of medications [men (OR 0.88, 95 % CI 0.84-0.93, p<0.001); women (OR 0.86, 95 % CI 0.82-0.91, p<0.001)]. All these associations remained robust following adjustments. Strong relationships were seen in both sexes between PA and taking medication for disorders of the central nervous system and gastrointestinal system, with relationships generally stronger in men. We have observed relationships between comorbid medical history and medication use with physical activity in a cohort of community dwelling older adults. These highlight the need to consider medical history when considering how best to optimize PA in older adults.

## Introduction

In recent decades a research literature has emerged showing a multitude of health benefits associated with frequent participation in physical activity. These include a risk reduction of developing type 2 diabetes mellitus (Smith et al., 2016[16]) and cardiovascular diseases (Lear et al., 2017[12]) and regular physical activity has also been shown to have a protective effect on depression risk (Gianfredi et al., 2020[9]). These findings are paramount in an aging society where the general population is projected to be living longer (Kontis et al., 2017[11]), and this increases the likelihood of developing age-related conditions and chronic conditions (Dhalwani et al., 2016[5]; Fortin et al., 2005[8]). Physical activity has been steadily promoted in the public health agenda by the World Health Organization (WHO, 2018[23]). In the 2015 World Report on Aging and Health, physical activity was highlighted as a key component to the concept of “healthy ageing” with particular focus on the derived benefits from physical activity participation in the older adult population (WHO, 2015[24]). Despite this drive for healthy aging the uptake of physical activity remains low in older adults (Salman and Sellami, 2019[14]). 

Multimorbidity has been linked to decreased physical activity in the general population (Chudasama et al., 2019[1]; Cimarras-Otal et al., 2014[2]) and some specific conditions such as chronic obstructive pulmonary disease (Mantoani et al., 2017[13]; Sánchez Castillo et al., 2020[15]) and inflammatory rheumatic conditions have been specifically identified as being a risk factor for low levels of activity (Cook et al., 2018[3]). However, data on the relationship between medical history and physical activity in older adults aged 80 years and above remain sparse with most studies involving younger participants aged 50-60 years and over, with far fewer participants at much advanced ages (Dhalwani et al., 2016[5]; Steeves et al., 2019[17]; Vancampfort et al., 2018[21]). For example, a cross-sectional study focusing on six low- and middle-income countries (China, Ghana, India, Mexico, Russia, South Africa) found participants aged ≥65 years old in the lowest tertile of activity had 1.28 times higher odds of physical multimorbidity than those in the highest tertile (Vancampfort et al., 2018[21]). In the UK, in the English Longitudinal Study of Ageing, researchers found an inverse dose response between levels of physical activity and multimorbidity in participants aged ≥50 years (Dhalwani et al., 2016[5]). 

In the present study we sought to explore, in a cohort of community-dwelling older adults in the UK in their eighth and ninth decades, whether self-report of chronic health conditions, medications, and medicated systems was associated with self-reported levels of physical activity. An understanding of such relationships may help clinicians caring for older adults living with several medical conditions, as it may allow focussed intervention in those at greatest risk.

## Methods

The Hertfordshire Cohort Study (HCS) is a population-based sample of 2997 individuals born in Hertfordshire between 1931 and 1939 and who still lived there in 1998-2004, when they were first recruited in order to study the relationship between growth in infancy and the subsequent risk of adult diseases (Syddall et al., 2005[18], 2019[19]). In 2014-2016, 1097 HCS participants completed postal questionnaires, which included questions on anthropometry, lifestyle, time spent doing physical activity in the past week, and their current health. 

Medical history included information on diagnosed conditions and medications they had been prescribed. Participants recruited for this study were selected from the total cohort sample, based on geographical location only. The participants were asked 'Do you have or have you ever had any of the following diseases?' A list of 12 common chronic condition categories were listed in the questionnaire, as follows: chronic non-specific lung disease (asthma, chronic bronchitis or pulmonary emphysema), cardio-vascular disease, peripheral arterial disease, diabetes mellitus, stroke, cancer, osteoporosis, rheumatoid/osteoarthritis, chronic liver or kidney disease, anorexia nervosa, overactive thyroid/parathyroid gland, and coeliac disease or malabsorption. 

Participants were asked to provide details of any medications they were currently taking. Medications were grouped according to the system medicated: cardiovascular, respiratory, gastro-intestinal, endocrine, central nervous system, malignant disease and immunosuppression, nutrition and blood, musculoskeletal and joint disease, eye, ear, nose, skin, genito-urinary tract and miscellaneous. Physical activity was assessed asking participants how much time, in the past week, they had spent taking part in various activities, namely: aerobics, aqua aerobics, playing badminton, playing bowls, cycling, dancing, playing football or hockey, light gardening (e.g. pruning, watering), heavy gardening (e.g. digging, mowing), playing golf, exercising at a gym, hiking, doing housework, jogging or running, snow skiing, playing squash, swimming, playing tennis, practicing tai chi/yoga/Pilates, walking, practicing water sports (e.g. windsurfing), and practicing any other activity not listed. Answers were recorded as <1 hour, 1-2 hours, 2-4 hours, and >4 hours, and scored as 1, 2, 3, and 4 respectively. These were then summed to generate an activity score, and based on the tertiles of the activity score, the participants were assigned to three different physical activity groups: low activity (scores <5), medium activity (scores ≥5 and <10), and high activity (scores ≥10).

The questionnaire also recorded several demographic and lifestyle factors. Participants were asked to self-report measurements of height and weight that were used to calculate body mass index (BMI) and smoker status was categorized as never smoked, ex-smoker or current smoker based on the following two questions “Are you a current smoker?” and “Have you ever smoked regularly?” Participants were asked whether they drank different types of alcohol (beer, wine, spirits, etc.) in the past week and how much they drank each time. This was used to estimate their alcohol consumption in units per week. Lastly, participants were asked whether, in the past year, they had any falls and if so how many.

## Statistical Analysis

Descriptive statistics for continuous variables were expressed as mean and standard deviation (SD) or median and interquartile range (IQR) as appropriate. Categorical variables were expressed as frequencies and percentages. Differences between men and women were assessed using Student's t-tests, Mann-Whitney tests or Pearson's χ^2^ tests, as appropriate. Ordinal logistic regression analyses were used to examine the associations between the number of chronic conditions, number of medications, and systems medicated and physical activity outcome (defined as low, medium or high activity). The regression analyses were undertaken with and without adjusting for age, BMI, smoker status, alcohol consumption. Our analyses were repeated after additional adjustment for falls in the last year. The results of the regression analyses are presented as odds ratios (OR) and 95 % confidence intervals (CI). A p-value of ≤0.05 was considered to be statistically significant. One of the models (relationships between respiratory disease and PA) breached the assumption of linearity between categories and is therefore not reported. The analyses were conducted using Stata version 16.

## Results

Data were available for 1097 participants, 555 men and 542 women. Table 1(Tab. 1) shows the demographic characteristics of the participants. The mean (SD) age of participants in the study was 80.2 (2.7) years for men and 80.2 (2.6) for women, with BMIs differing very little between the sexes. More men (10.5 %) than women (8.4 %) reported that they were not taking any medications. Women were more likely to have fallen in the past year than men, 62.7 % of female participants reported no falls in the past 12 months compared to 66.7 % of male participants. These differences, however, were not statistically significant. A higher proportion of men were in the high activity score group (33.7 %) than women (24 %). 20.8 % of female participants reported having no medical conditions, while 22.6 % of men said they did not have any of the conditions listed. A higher percentage of men, 10.5 % reported they took no medication compared to 8.4 % of women.

We explored the relationship between the number of comorbidities, number of medications, and system medicated with physical activity (Table 2(Tab. 2)). The number of chronic conditions was significantly associated with lower levels of physical activity: both men (OR 0.73, 95 % CI 0.63-0.84, p<0.001) and women (OR 0.74, 95 % CI 0.64-0.86, p<0.001) such that those participants with a higher number of chronic conditions had a lower odds of being in a higher activity score category compared to being in a lower category after adjustment for other factors i.e. assuming these are held constant. Similarly, being prescribed a higher number of medications was associated with a lower odds of being in a higher activity score category in both men (OR 0.88, 95 % CI 0.84-0.93, p<0.001) and women (OR 0.86, 95 % CI 0.82-0.91, p<0.001). All these associations remained robust following adjustment for age, BMI, smoker status, alcohol consumption (all *p* ≤0.001).

Table 2(Tab. 2) also shows relationships between system medicated and physical activity. Strong relationships were seen in both sexes between PA and taking medication for disorders of the central nervous system and gastrointestinal system, with statistical significance maintained after adjustments. In men we noted those taking medications for the musculoskeletal and joint, nutrition and blood and endocrine systems had significantly lower odds of being in a higher activity score category than being in a lower category. 

Finally we performed our analyses before and after adjustment for number of falls in the last year. This did not materially affect our results (data not shown). 

## Discussion

In this study we have reported high rates of community dwelling older adults living with chronic health conditions in a UK cohort. We have also found that those individuals with higher number of comorbidities and number of medications were less likely to be active. These relationships were particularly marked in men. These data are significant as they represent a rare investigation of much older community dwelling adults.

The term multimorbidity has been used to decribe the presence of multiple chronic conditions but there remains much heterogeneity in the definition used in research literature (Johnston et al., 2018[10]), however the common cut off is the presence of two or more conditions (Johnston et al., 2018[10]), and this is echoed by NICE (National Institute for Health and Care Excellence) where its guideline defines multimorbidity as “the presence of two or more long-term health conditions”. Using this definition, the presence of multimorbidity in our sample was 37.8 % in men and 43.7 % in women. The prevalence of multimorbidity reported elsewhere varies depending on the geographical location of studies. For example, the UK Biobank reported the prevalence to be 19 % in a cohort of 502,643 participants aged 40 to 69 years (Zemedikun et al., 2018[25]), whereas the Scottish Health Survey found the prevalence of two or more comorbidities to be 44 % but this is likely to reflect the older age range of the participants for the Scottish Health Survey, 65 years and over (Salman and Sellami, 2019[14]). Given the age range of our study it is difficult to make a direct comparison with current literature, however the presence of chronic conditions was linked to lower odds of being physically active in our cohort of older adults, is in keeping with other studies involving participants younger than our study cohort (Chudasama et al., 2019[1]; Dhalwani et al., 2016[5]; Vancampfort et al., 2018[21]). 

We found that overall taking more medications was associated with a lower chance of being in a higher activity score category compared to being in a lower category. These data are consistent with a study of a German older adult cohort, aged 65-94 years, that found those participants in the lower tertile compared to the higher tertile of physical activity were more likely to be on >4 medications (OR: 1.64, 95 % CI: 1.05- 2.56, P=0.031) (Volaklis et al., 2018[22]). In another work, in a US cohort, aged 49-79 years, an increased number of prescription medications (≥5 medications) was associated cross-sectionally with decreased objectively measured physical activity compared to those who did not meet the medication criterion, although this cohort was recruited on the basis of having knee osteoarthritis (Thanoo et al., 2020[20]). 

There are likely to be a number of mechanisms at play here including the underlying medical condition and as well as the potential side effects experienced from taking the medications themselves that may result in lower participation in physical activity. Another possible mechanism is falls risk, as several of the conditions studied may increase falls risk directly or be associated with medication which can lead to enhanced falls risk. However, we undertook our analysis before and after adjustment for falls, which did not change our findings, suggesting that this was not a major modifier of PA in this group.

In this study we were able to explore the relationship between the use of various medications and physical activity in older adults, in particular the relationship between different systems medicated and physical activity which has not been well studied previously. We saw some differences between men and women for these relationships; in general, these were stronger in men than women, an observation that would be interesting to explore further in future work. For example, prescription of central nervous system medication was a very strong predictor of reduced physical activity. Such associations might be anticipated - for example, Parkinson's' disease is a chronic progressive neurological condition that leads to immobility which in turns impact on participation of physical activity (Ellis and Motl, 2013[7]). 

There are some limitations to this study. Our study population may not be entirely representative of the wider UK population, since all recruited participants were born in the county of Hertfordshire, and were all Caucasian. Nevertheless, it has been previously demonstrated that the HCS is representative of the general population with regard to anthropometric body build and lifestyle factors, such as smoking and alcohol intake, which was in line with data found in the European Investigation into Cancer and Nutrition Cohort (EPIC) (Dik et al., 2014[6]). Comorbidities were self-reported and therefore recall bias cannot be ruled out, however we used medication prescribed to validate the self-reported conditions. We do not know how long the medication had been taken for, nor indeed whether subjects were compliant with their therapy. We also have no information on severity of disease. Physical activity was self reported, but has previously been validated (Deere et al., 2016[4]). Finally, fewer subjects just had one system medicated making it harder to look at relationships between individual comorbidities and PA.

In conclusion we have observed relationships between comorbidities and medication taken with physical activity in a cohort of community dwelling older adults aged 77 years and over. The prevalence of multimorbidity is high in the older adult population, and this is likely to increase as the population lives longer. Considering which medications are being prescribed to older adults, and the medical conditions underlying these prescriptions, might help guide clinicians caring for older patients to discuss PA levels, and might also inform a research agenda that considers interventions in individual patients groups.

## Notes

Gregorio Bevilacqua and Jean Zhang contributed equally as first author.

## Declaration

### Acknowledgments

We thank the Hertfordshire Cohort study participants who made this work possible. This work was funded by the Medical Research Council. FL and JZ are supported by the NIHR Southampton Biomedical Research Centre, and the University of Southampton.

### Conflict of interest

Professor Cyrus Cooper has received lecture fees and honoraria from Amgen, Danone, Eli Lilly, GSK, Kyowa Kirin, Medtronic, Merck, Nestlé, Novartis, Pfizer, Roche, Servier, Shire, Takeda and UCB outside of the submitted work. Drs. Faidra Laskou and Jean Zhang are supported by the NIHR Southampton Biomedical Research Centre, and the University of Southampton. Professor Elaine Dennison has received lecture fees and honoraria from UCB, Pfizer, Lilly and Viatris outside of the submitted work. Dr. Nicholas Fuggle has received travel and educational bursaries from Pfizer and Lilly. Drs. Gregorio Bevilacqua and Millie Parsons declare no conflicts of interest.

## Figures and Tables

**Table 1:**
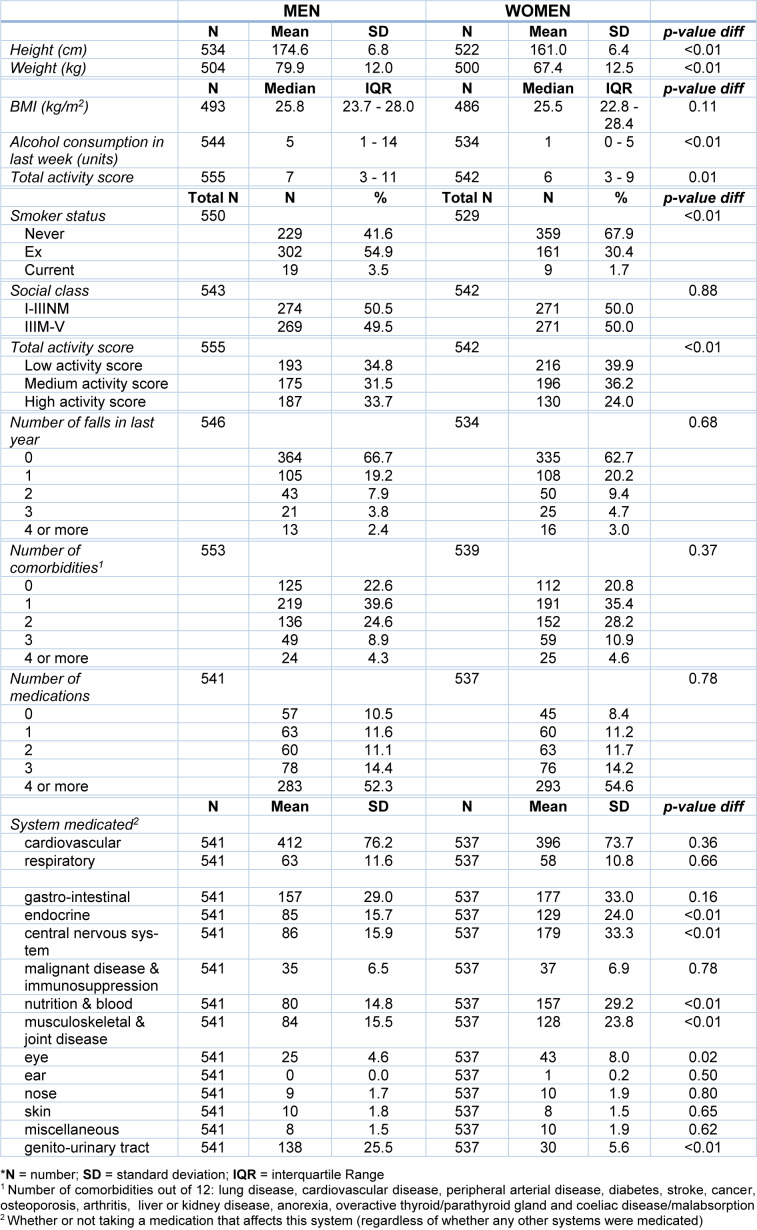
Participants' characteristics*

**Table 2:**
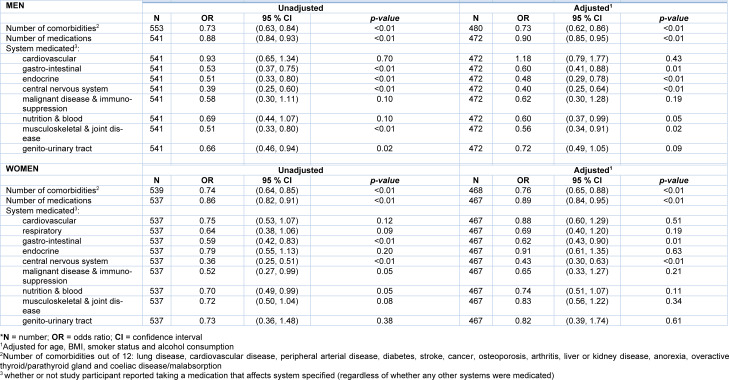
Number of comorbidities, number of medications and system medicated as explanatory variables for activity score (low, medium, high) - Odds ratios are the odds of being in a higher activity score category compared to being in a lower category*
